# Severe Metabolic Acidosis, Acute Renal Failure, and Delayed Paralysis Leading to Respiratory Depression due to Manganese ethylene-bis-dithiocarbamate

**DOI:** 10.5152/TJAR.2023.21596

**Published:** 2023-02-01

**Authors:** Dilek Güven Taymez, Özge Telci Çaklılı, Hande Gürbüz

**Affiliations:** 1 Nephrology Clinic, Kocaeli State Hospital, Kocaeli, Turkey; 2 Department of Endocrinology and Metabolism, İstanbul University Faculty of Medicine, İstanbul, Turkey; 3 University of Health Sciences, Bursa Yüksek İhtisas Training and Research Hospital, Bursa, Turkey

**Keywords:** Acute kidney injury, clinical toxicology,, maneb, oxidative stress, paralysis, pesticides, toxic environmental substances

## Abstract

Maneb is a widely used agricultural fungicide, which can lead to parkinsonism due to its neurotoxic effects on the dopaminergic system after chronic low-dose exposure. Previous acute human maneb poisoning cases occurred with low-dose maneb exposure through the dermal route causing renal failure. This report presents a case of acute renal failure and delayed paralysis due to ingestion of a large dose of maneb for a suicide attempt. A 16-year-old female patient was admitted to the emergency room because of drinking almost a whole bottle of maneb (400 mL [2 g L^−1^]) about 2 hours before. The patient was transferred to the intensive care unit with severe metabolic acidosis and renal failure. On the 4th day in intensive care unit, although the severe acidosis was resolved with haemodialysis, the patient was intubated because of ascending muscle weakness and dyspnoea. After staying in the intensive care unit for 9 days and in the nephrology ward for 2 weeks, the patient was discharged well from the hospital with no further need for haemodialysis but a persistent bilateral drop foot. One year after the event, renal functions were normal, and motor function in the lower extremities improved entirely.

**Main Points**

Maneb contains manganese as a metal moiety, a carbamate organic backbone, and a thiol group.
Maneb can cause Parkinsonism, usually following a chronic low-dose exposure through the dermal route.
Acute poisoning cases can present kidney failure and metabolic acidosis.
Acute high-dose exposure through the oral route can be related to intermediate syndrome due to the carbamate ingredient.

## Introduction

Manganese ethylene-bis-dithiocarbamate (maneb) is a widely used agricultural fungicide. Previous human poisoning reports include accidental exposure to a low amount of this pesticide, usually through the dermal route. This report presents a case of acute renal failure and delayed paralysis due to ingestion of a large dose of maneb.

## Case Presentation

A 16-year-old female patient was brought to the emergency room due to a suicide attempt with ingesting almost a whole bottle of maneb (approximately 400 mL [2 g L^−1^]) about 2 hours before. The medical history was unremarkable for any other disease, and the patient was not under any medication. The patient was oriented, cooperative, and agitated. The heart rate was 112 per minute (sinus rhythm with no remarkable electrocardiogram [ECG] findings), blood pressure was 140/90 mmHg, and CK-MB and troponin-I levels stayed at the upper limits of maximum levels. The patient had no fever, nausea, vomiting, or diarrhoea. There were no cholinergic symptoms such as excessive salivation, bronchial secretion, and lacrimation. Additionally, no rigidity, dystonia, or paraesthesia was detected in neurologic evaluation. Laboratory analysis revealed severe metabolic acidosis (pH: 6.9, BE: −21.4 mEq L^−1^, HCO_3_: 9.9 mEq L^−1^, Lac: 15), and the creatinine level was 2.9 mg dL^−1^.

The patient was referred to the intensive care unit (ICU), then the metabolic acidosis dramatically resolved after intermittent bicarbonate-based haemofiltration for 4-hours per day on the consequent 3 days. The urine output was adequate, and no fluid removal was prescribed. On further laboratory investigations, free T3 levels were slightly elevated, and free T4 and thyroid-stimulating hormone levels remained normal, blood albumin was 2.3 g dL^−1^, proteinuria was 1.5 g day^−1^, and there was microscopic haematuria (+++). On the 4th day of ICU follow-up, the patient was intubated because of ascending muscle weakness and dyspnoea, with no infection or haemodynamic instability findings. The neurologic evaluation revealed that the deep tendon reflexes in the lower extremities were diminished. Cranial neurologic pathologies were excluded through cranial imaging; however, electromyography could not be performed in the ICU. The patient was weaned from the mechanical ventilator after 5 days of ventilation and then discharged to the ward. Intermittent haemodialysis continued for an additional week (3 days per week). The patient was discharged well from the hospital without further haemodialysis; however, bilateral drop-foot persisted. One year after the event, renal functions were normal, and motor function in the lower extremities was improved entirely. Parental consent was obtained to publish this report.

## Discussion

### General Information

Maneb is generally absorbed by oral and dermal routes.^1^ It mainly accumulates in the thyroid gland, kidney, and heart, and it can also cause respiratory paralysis leading to death.^2^ Acute intoxication in humans is rare except for limited inflammatory reactions on the skin and mucous membranes. Due to its high lipo-solubility, maneb passes through the blood–brain barrier and is used for creating Parkinson’s animal models.^3^ There is no known specific treatment for acute maneb intoxication. The only proposed method for treatment is to maintain systemic functions.^1^

According to the cell-line and animal studies, the primary pathophysiology of maneb poisoning has been shown to produce robust reactive oxygen species, resulting in mitochondrial dysfunction due to oxidative stress.^4,5^ With this mechanism, maneb leads to parkinsonism with its neurotoxic effect on dopaminergic and GABAergic neurons and kidney dysfunction with its nephrotoxic effect on nephrons.^6-9^ At the chemical base, maneb contains manganese as a metal moiety, a thiol group, and carbamate as an organic backbone.^10^ However, *in vitro* and *in vivo* studies do not totally explain in detail the whole mechanism in human exposures but provide valuable insights.

### Differential Diagnosis

The possible differential diagnoses were food poisoning, illegal chemical substance abuse, myasthenia gravis, Guillain Barre syndrome, intracranial pathologies, and other reasons for acute renal insufficiency. Lack of gastrointestinal symptoms and infection findings in the patient and her parents, who had eaten the same foods, ruled out food poisoning. Although we did not have electromyography, the lack of preceding history of any infectious disease and vaccination, lack of ophthalmoplegia, and normal cranial imaging ruled out Guillain Barre syndrome, myasthenia gravis, and intracranial pathologies. The use of illegal substances was excluded. Furthermore, a clear anamnesis of ingestion of a definite agricultural substance led us to focus on maneb intoxication.

### Discussion of the Case in the View of Previous Literature

Acute renal toxicity of maneb in humans has been mentioned only in 2 previous case reports.^11,12^ Both reported human cases of acute maneb intoxication had only dermal contact with the substance. The first case by Koizumi et al^11^ was a 62-year-old male patient who was admitted to the hospital with hoarseness and muscle weakness; then, an acute renal failure with proteinuria, microscopic haematuria, and metabolic acidosis had developed. There was ST-segment depression on ECG suggesting myocardial ischaemia, which was attributed to a hypotensive vagotonic state elicited by maneb and the use of fluorouracil. The cardiac symptoms and metabolic acidosis resolved with haemodialysis. In our patient, we did not observe any hypotensive period, there were no ECG findings, and cardiac markers showed no further elevation.

The second patient by de Carvalho et al^12^ had nausea, vomiting, and diarrhoea on the following day of exposure. The patient was admitted to the hospital with peripheral oedema and reduced diuresis. A persistent nephrotic syndrome was observed that was treated with steroids but not haemodialysis. Additionally, the patient’s renal biopsy showed severe tubular lesions mainly affecting the proximal convoluted tubules (cell swelling, necrosis, vacuolisation, and disappearance of the brush border). Our case’s clinical presentation was not in accordance with nephrotic syndrome. Although lactic acidosis was not mentioned in the previous 2 cases (but metabolic acidosis was in both), this can be the manifestation of human maneb poisoning.

Maneb is thought to cause muscle weakness and respiratory paralysis; however, this undesirable result’s mechanism is unclear.^1,2,11^ The case presented by de Carvalho et al^12^ had no neurologic findings, while case of Koizumi et al^11^ showed hoarseness and knee and ankle jerk was exaggerated. Our patient had no extrapyramidal system findings; however, muscle weakness with respiratory inadequacy requiring mechanical ventilation developed on the 4th day. Because the patient had no metabolic or autoimmune disease and that there was a massive exposure to maneb, we hypothesized that the ascending muscle weakness in this patient might be the delayed effect of the organic part – the carbamate ingredient of maneb.^13^ The clinical findings were consistent with delayed neuropathy; thus, the diagnosis was likely to be the intermediate syndrome.^13,14^ Pralidoxime was not initiated because of the lack of cholinergic findings, and that pralidoxime was found to exacerbate the acetylcholine inactivation when administered in carbamate poisoning.^15^

## Conclusion

There is limited data regarding acute high-dose maneb intoxication in humans. Maneb is related to Parkinsonism in low-dose chronic exposure. However, its nephrotoxic effect becomes prominent in acute exposure. Furthermore, acute high-dose intoxication may lead to intermediate syndrome due to carbamate ingredient.
